# Tetra­ethyl­ammonium hexa­cyanidoferrate(III) bis­(diaqua­{6,6′-dimeth­oxy-2,2′-[*o*-phenyl­enebis(nitrilo­methyl­idyne)]diphenolato}manganese(III))–methanol–ethanol (1/2/2)

**DOI:** 10.1107/S1600536809037490

**Published:** 2009-10-17

**Authors:** Ting-Ting Wang, Ji-Min Xie

**Affiliations:** a School of Chemistry and Chemical Engineering, Jiangsu University, Zhenjiang 212013, People’s Republic of China

## Abstract

In the title compound, (C_8_H_20_N)[Mn(C_22_H_18_N_2_O_4_)(H_2_O)_2_][Fe(CN)_6_]·2CH_3_OH·2C_2_H_5_OH or [NEt_4_][Mn(3-Meosalophen)(H_2_O)_2_]_2_[Fe(CN)_6_]·2CH_3_OH·2C_2_H_5_OH, the asymmetric unit consists of one half of an [NEt_4_]^+^ cation disordered around a twofold axis, the [Mn(3-Meosalophen)(H_2_O)_2_]^+^ coordination cation, one half of a *C*_2_ symmetric [Fe(CN)_6_]^3−^ anion and disordered methanol and ethanol solvent mol­ecules that are equally populated at two different sites. The Mn^III^ atom chelated by the 3-Meosalophen ligand adopts a slightly distorted MnN_2_O_4_ octa­hedral geometry with the coordination completed by two water mol­ecules. The [Mn(3-Meosalophen)(H_2_O)_2_]^+^ cations, [Fe(CN)_6_]^3- ^anions and solvent mol­ecules are connected into a zigzag chain through hydrogen-bonding inter­actions.

## Related literature

For related structures, see: Li *et al.* (2001 ▶). For the preparation of the precursors, [Mn(3-Meosalphen)(H_2_O)(CH_3_OH)]ClO_4_ and [NEt_4_]_3_[Fe(CN)_6_], see: Matsumoto *et al.* (1988 ▶); Mascharak *et al.*(1986 ▶). 
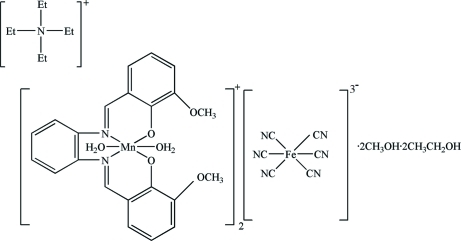


## Experimental

### 

#### Crystal data

(C_8_H_20_N)[Mn(C_22_H_18_N_2_O_4_)(H_2_O)_2_][Fe(CN)_6_]·2CH_4_O·2C_2_H_6_O
*M* *_r_* = 1429.15
Monoclinic, 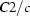

*a* = 24.83 (2) Å
*b* = 12.467 (11) Å
*c* = 22.915 (19) Å
β = 98.077 (12)°
*V* = 7024 (10) Å^3^
*Z* = 4
Mo *K*α radiation
μ = 0.63 mm^−1^
*T* = 296 K
0.22 × 0.22 × 0.15 mm

#### Data collection

Rigaku Mercury CCD diffractometer
Absorption correction: multi-scan (*CrystalClear*; Rigaku, 2002 ▶) *T*_min_ = 0.871, *T*_max_ = 0.910
29161 measured reflections
8077 independent reflections
4987 reflections with *I* > 2σ(*I*)
*R*_int_ = 0.077

#### Refinement

*R*[*F* ^2^ > 2σ(*F* ^2^)] = 0.048
*wR*(*F* ^2^) = 0.129
*S* = 1.05
8077 reflections
483 parameters
7 restraints
H-atom parameters constrained
Δρ_max_ = 0.68 e Å^−3^
Δρ_min_ = −0.61 e Å^−3^

### 

Data collection: *CrystalClear* (Rigaku, 2002 ▶); cell refinement: *CrystalClear*; data reduction: *CrystalClear*; program(s) used to solve structure: *SHELXS97* (Sheldrick, 2008 ▶); program(s) used to refine structure: *SHELXL97* (Sheldrick, 2008 ▶); molecular graphics: *DIAMOND* (Brandenburg & Putz, 2006 ▶) and *SHELXTL* (Sheldrick, 2008 ▶); software used to prepare material for publication: *SHELXL97*.

## Supplementary Material

Crystal structure: contains datablocks I, global. DOI: 10.1107/S1600536809037490/gk2224sup1.cif

Structure factors: contains datablocks I. DOI: 10.1107/S1600536809037490/gk2224Isup2.hkl

Additional supplementary materials:  crystallographic information; 3D view; checkCIF report

## Figures and Tables

**Table 1:** Selected bond lengths (Å)

Mn1—O2	1.880 (2)
Mn1—O1	1.884 (2)
Mn1—N5	1.996 (2)
Mn1—N4	1.996 (3)
Mn1—O2*W*	2.210 (3)
Mn1—O1*W*	2.274 (2)
Fe1—C24	1.945 (3)
Fe1—C23	1.953 (3)
Fe1—C25	1.958 (4)

**Table 2:** Hydrogen-bond geometry (Å, °)

*D*—H⋯*A*	*D*—H	H⋯*A*	*D*⋯*A*	*D*—H⋯*A*
O1*W*—H1*C*⋯O3^i^	0.85	2.14	2.959 (4)	162
O1*W*—H1*C*⋯O1^i^	0.85	2.37	2.948 (3)	125
O1*W*—H1*D*⋯O4^i^	0.85	2.14	2.929 (3)	153
O1*W*—H1*D*⋯O2^i^	0.85	2.25	2.901 (3)	134
O2*W*—H2*C*⋯O6^ii^	0.85	1.88	2.698 (4)	160
O2*W*—H2*D*⋯O5^iii^	0.85	1.91	2.751 (4)	168
O5—H5*B*⋯N3	0.87	2.08	2.934 (5)	166
O6—H6*B*⋯N2	0.86	1.88	2.739 (4)	173

Symmetry codes: (i) 
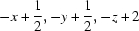
; (ii) 
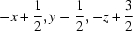
; (iii) 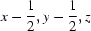.

**Fig. 1.**
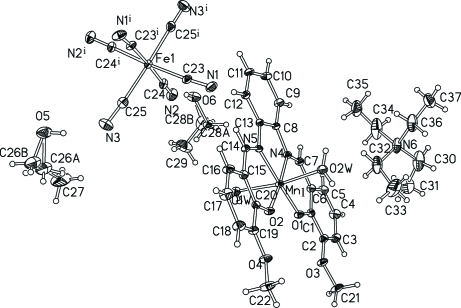
The structure of the title compound with the atomic labels and 30% probability displacement ellipsoids for non-hydrogen atoms. Disorder of the [NEt4]+ cation is not shown. Symmetry code: (i) 1 - x,y, 1.5 - z.

**Fig. 2.**
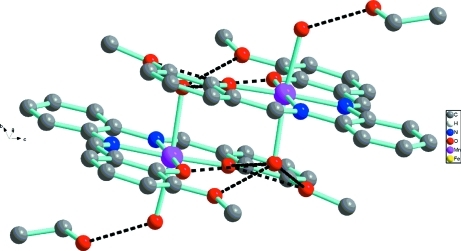
The intermolecular O—H··· O hydrogen bonding interactions connecting the [Mn(3-Meosalophen)(H2O)2]+ units into a dimer. H atoms have been omitted for clarity and hydrogen bonds are shown as dashed line.

**Fig. 3.**
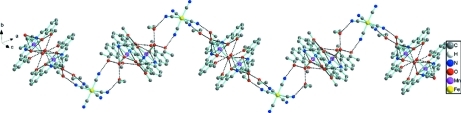
A view of a one-dimensional zigzag chain. H atoms have been omitted for clarity and hydrogen bonds are shown as dashed line.

## supplementary crystallographic information

### Comment

Manganese(III) Schiff base complexes and [NEt_4_]_3_[Fe(CN)_6_] are often used as precursors to construct magnetic compounds, which demonstrate various networks and topologies. The asymmetric unit of of the title compound comprises one half of [NEt_4_]^+^ cation (disordered), one [Mn(3-Meosalophen)(H_2_O)_2_]^+^ cation, one half of [Fe(CN)_6_]^3-^ anion and two halves of methanol and ethanol solvent molecules. Both the Mn(III) atom and Fe(III) atom exhibit a slightly distorted octahedral coordination geometry (Fig.1). Adjacent [Mn(3-Meosalophen)(H_2_O)_2_]^+^ units are aggregated into a dimer through O—H···O hydrogen bonding interactions as well as *π*-*π* interactions (Fig.2). These dimers and coordination anions are further connected into a one-dimensional zigzag chain through O—H···N hydrogen bonds involving solvent molecules (Fig.3). The chains are further packed into a three-dimensional framework through weak intermolecular interactions.

### Experimental

A solution of [Mn(3-Meosalphen)(H_2_O)(CH_3_OH)]ClO_4_ (0.1094 g) in 1:1 (*v*/*v*) methanol-acetonitrile (30 ml) was added to a solution of [NEt_4_]_3_[Fe(CN)_6_] (0.1205 g) in ethanol (30 ml) at room temperature. The resulting solution was filtered and the filtrate was kept in the dark. Black block crystals of the title compound were obtained after a week (yield 85%).

### Refinement

All the H atoms were placed at calculated positions (C—H 0.93–0.98 Å, O—H 0.85-0.87 Å), and treated as riding atoms with *U*_iso_(H)=1.2*U*_eq_(C), *U*iso(H)=1.5*U*_eq_(O).

### Crystal data

**Table:**

(C_8_H_20_N)[Mn(C_22_H_18_N_2_O_4_)(H_2_O)_2_][Fe(CN)_6_]·2CH_4_O·2C_2_H_6_O	*F*(000) = 2996
*M**_r_* = 1429.15	*D*_x_ = 1.351 Mg m^−^^3^
Monoclinic, *C*2/*c*	Mo *K*α radiation, λ = 0.71073 Å
Hall symbol: -C 2yc	Cell parameters from 6244 reflections
*a* = 24.83 (2) Å	θ = 2.3–27.5°
*b* = 12.467 (11) Å	µ = 0.63 mm^−^^1^
*c* = 22.915 (19) Å	*T* = 296 K
β = 98.077 (12)°	Prism, black
*V* = 7024 (10) Å^3^	0.22 × 0.22 × 0.15 mm
*Z* = 4	

### Data collection

**Table:**

Rigaku Mercury CCD diffractometer	8077 independent reflections
Radiation source: fine-focus sealed tube	4987 reflections with *I* > 2σ(*I*)
graphite	*R*_int_ = 0.077
Detector resolution: 0 pixels mm^-1^	θ_max_ = 27.6°, θ_min_ = 2.3°
ω scans	*h* = −32→32
Absorption correction: multi-scan (*CrystalClear*; Rigaku, 2002)	*k* = −15→16
*T*_min_ = 0.871, *T*_max_ = 0.910	*l* = −29→29
29161 measured reflections	

### Refinement

**Table:**

Refinement on *F*^2^	Primary atom site location: structure-invariant direct methods
Least-squares matrix: full	Secondary atom site location: difference Fourier map
*R*[*F*^2^ > 2σ(*F*^2^)] = 0.048	Hydrogen site location: inferred from neighbouring sites
*wR*(*F*^2^) = 0.129	H-atom parameters constrained
*S* = 1.05	*w* = 1/[σ^2^(*F*_o_^2^) + (0.05*P*)^2^ + 6*P*] where *P* = (*F*_o_^2^ + 2*F*_c_^2^)/3
8077 reflections	(Δ/σ)_max_ = 0.001
483 parameters	Δρ_max_ = 0.68 e Å^−^^3^
7 restraints	Δρ_min_ = −0.61 e Å^−^^3^

### Special details

**Table:**

Geometry. All e.s.d.'s (except the e.s.d. in the dihedral angle between two l.s. planes) are estimated using the full covariance matrix. The cell e.s.d.'s are taken into account individually in the estimation of e.s.d.'s in distances, angles and torsion angles; correlations between e.s.d.'s in cell parameters are only used when they are defined by crystal symmetry. An approximate (isotropic) treatment of cell e.s.d.'s is used for estimating e.s.d.'s involving l.s. planes.
Refinement. Refinement of *F*^2^ against ALL reflections. The weighted *R*-factor *wR* and goodness of fit *S* are based on *F*^2^, conventional *R*-factors *R* are based on *F*, with *F* set to zero for negative *F*^2^. The threshold expression of *F*^2^ > σ(*F*^2^) is used only for calculating *R*-factors(gt) *etc*. and is not relevant to the choice of reflections for refinement. *R*-factors based on *F*^2^ are statistically about twice as large as those based on *F*, and *R*- factors based on ALL data will be even larger.

### Fractional atomic coordinates and isotropic or equivalent isotropic displacement parameters (Å^2^)

**Table:**

	*x*	*y*	*z*	*U*_iso_*/*U*_eq_	Occ. (<1)
Mn1	0.225696 (15)	0.19335 (4)	0.900599 (18)	0.02057 (13)	
N4	0.19214 (9)	0.2894 (2)	0.83582 (10)	0.0222 (6)	
N5	0.28072 (8)	0.17122 (19)	0.84607 (10)	0.0200 (5)	
O1	0.17142 (7)	0.22371 (17)	0.94799 (8)	0.0257 (5)	
O1W	0.27767 (7)	0.33754 (17)	0.93263 (9)	0.0270 (5)	
H1C	0.3072	0.3103	0.9495	0.041*	
H1D	0.2583	0.3811	0.9492	0.041*	
O2	0.26287 (7)	0.10405 (17)	0.95897 (8)	0.0242 (5)	
O2W	0.17979 (9)	0.05630 (19)	0.85739 (10)	0.0389 (6)	
H2C	0.1806	0.0453	0.8209	0.058*	
H2D	0.1710	0.0078	0.8803	0.058*	
O3	0.10898 (8)	0.23257 (19)	1.02928 (9)	0.0335 (6)	
O4	0.29094 (8)	−0.02186 (18)	1.04843 (9)	0.0334 (6)	
C1	0.12920 (10)	0.2894 (2)	0.93726 (13)	0.0237 (7)	
C2	0.09377 (11)	0.2957 (3)	0.98106 (13)	0.0281 (7)	
C3	0.04880 (12)	0.3622 (3)	0.97292 (15)	0.0364 (8)	
H3A	0.0254	0.3644	1.0012	0.044*	
C4	0.03832 (12)	0.4260 (3)	0.92262 (15)	0.0397 (9)	
H4A	0.0083	0.4714	0.9180	0.048*	
C5	0.07180 (12)	0.4224 (3)	0.87996 (15)	0.0352 (8)	
H5A	0.0647	0.4660	0.8468	0.042*	
C6	0.11749 (11)	0.3522 (3)	0.88629 (13)	0.0258 (7)	
C7	0.14969 (11)	0.3500 (3)	0.83889 (13)	0.0262 (7)	
H7A	0.1394	0.3962	0.8074	0.031*	
C8	0.22164 (11)	0.2927 (2)	0.78637 (12)	0.0222 (6)	
C9	0.20642 (12)	0.3505 (3)	0.73467 (13)	0.0302 (7)	
H9A	0.1743	0.3899	0.7297	0.036*	
C10	0.23945 (13)	0.3492 (3)	0.69047 (14)	0.0337 (8)	
H10A	0.2288	0.3864	0.6556	0.040*	
C11	0.28830 (12)	0.2927 (3)	0.69809 (13)	0.0293 (7)	
H11A	0.3109	0.2948	0.6690	0.035*	
C12	0.30333 (11)	0.2338 (3)	0.74860 (13)	0.0254 (7)	
H12A	0.3357	0.1953	0.7532	0.030*	
C13	0.26995 (10)	0.2317 (2)	0.79300 (12)	0.0210 (6)	
C14	0.32186 (10)	0.1053 (2)	0.85617 (12)	0.0231 (7)	
H14A	0.3447	0.1006	0.8274	0.028*	
C15	0.33504 (11)	0.0397 (2)	0.90729 (13)	0.0235 (7)	
C16	0.37990 (11)	−0.0311 (3)	0.90782 (14)	0.0312 (8)	
H16A	0.3998	−0.0313	0.8763	0.037*	
C17	0.39398 (12)	−0.0988 (3)	0.95406 (15)	0.0370 (9)	
H17A	0.4230	−0.1459	0.9536	0.044*	
C18	0.36498 (12)	−0.0981 (3)	1.00261 (15)	0.0342 (8)	
H18A	0.3752	−0.1438	1.0343	0.041*	
C19	0.32150 (11)	−0.0299 (3)	1.00317 (13)	0.0256 (7)	
C20	0.30518 (10)	0.0404 (2)	0.95564 (12)	0.0208 (6)	
C21	0.07320 (14)	0.2277 (3)	1.07371 (16)	0.0481 (10)	
H21A	0.0886	0.1812	1.1051	0.072*	
H21B	0.0384	0.2003	1.0565	0.072*	
H21C	0.0688	0.2983	1.0891	0.072*	
C22	0.30130 (15)	−0.0992 (3)	1.09498 (16)	0.0485 (10)	
H22A	0.2775	−0.0860	1.1238	0.073*	
H22B	0.3384	−0.0933	1.1132	0.073*	
H22C	0.2949	−0.1700	1.0791	0.073*	
Fe1	0.5000	0.20032 (5)	0.7500	0.02048 (15)	
N1	0.41520 (11)	0.0258 (2)	0.76940 (13)	0.0398 (7)	
N2	0.41795 (11)	0.3708 (2)	0.78020 (13)	0.0413 (8)	
N3	0.55820 (12)	0.2277 (3)	0.87850 (13)	0.0478 (8)	
C23	0.44759 (11)	0.0900 (3)	0.76426 (13)	0.0268 (7)	
C24	0.44855 (11)	0.3079 (3)	0.76920 (14)	0.0270 (7)	
C25	0.53541 (12)	0.2119 (3)	0.83165 (15)	0.0312 (8)	
O5	0.64242 (12)	0.3872 (2)	0.91758 (12)	0.0636 (8)	
H5B	0.6144	0.3442	0.9107	0.076*	
C26A	0.6553 (5)	0.3967 (12)	0.9782 (6)	0.068 (3)	0.50
H26A	0.6842	0.4482	0.9890	0.082*	0.50
H26B	0.6655	0.3282	0.9966	0.082*	0.50
C27	0.5956 (4)	0.4407 (8)	0.9956 (3)	0.100 (4)	0.50
H27A	0.5987	0.4517	1.0374	0.120*	0.50
H27B	0.5678	0.3884	0.9837	0.120*	0.50
H27C	0.5862	0.5072	0.9756	0.120*	0.50
C26B	0.6324 (4)	0.4138 (8)	0.9832 (3)	0.068 (3)	0.50
H26C	0.5954	0.4365	0.9830	0.102*	0.50
H26D	0.6565	0.4700	0.9990	0.102*	0.50
H26E	0.6391	0.3508	1.0072	0.102*	0.50
O6	0.34304 (10)	0.5328 (2)	0.76054 (11)	0.0492 (7)	
H6B	0.3679	0.4840	0.7643	0.059*	
C28A	0.3171 (8)	0.5434 (18)	0.8071 (9)	0.066 (7)	0.50
H28A	0.2866	0.5912	0.7960	0.080*	0.50
H28B	0.3028	0.4723	0.8128	0.080*	0.50
C29	0.3422 (5)	0.5759 (10)	0.8626 (4)	0.101 (4)	0.50
H29A	0.3162	0.5749	0.8899	0.152*	0.50
H29B	0.3562	0.6473	0.8601	0.152*	0.50
H29C	0.3716	0.5279	0.8761	0.152*	0.50
C28B	0.3076 (8)	0.5330 (15)	0.8069 (7)	0.035 (4)	0.50
H28C	0.2822	0.4746	0.8005	0.053*	0.50
H28D	0.2880	0.5996	0.8057	0.053*	0.50
H28E	0.3293	0.5251	0.8447	0.053*	0.50
N6	−0.0021 (11)	0.1619 (5)	0.7622 (8)	0.047 (4)	0.50
C30	−0.0566 (3)	0.1690 (8)	0.7791 (5)	0.083 (3)	0.50
H30A	−0.0815	0.1909	0.7452	0.100*	0.50
H30B	−0.0673	0.0985	0.7899	0.100*	0.50
C31	−0.0630 (7)	0.2493 (14)	0.8296 (6)	0.080 (4)	0.50
H31A	−0.0999	0.2483	0.8377	0.120*	0.50
H31B	−0.0390	0.2287	0.8644	0.120*	0.50
H31C	−0.0539	0.3203	0.8181	0.120*	0.50
C32	0.0420 (3)	0.1427 (8)	0.8130 (5)	0.065 (3)	0.50
H32A	0.0762	0.1328	0.7987	0.078*	0.50
H32B	0.0452	0.2058	0.8372	0.078*	0.50
C33	0.0319 (11)	0.0444 (18)	0.8536 (11)	0.086 (6)	0.50
H33A	0.0615	0.0390	0.8853	0.129*	0.50
H33B	0.0299	−0.0201	0.8305	0.129*	0.50
H33C	−0.0015	0.0539	0.8695	0.129*	0.50
C34	0.0129 (4)	0.2695 (7)	0.7327 (7)	0.087 (5)	0.50
H34A	−0.0177	0.2925	0.7053	0.104*	0.50
H34B	0.0189	0.3224	0.7634	0.104*	0.50
C35	0.0620 (6)	0.2682 (14)	0.7047 (6)	0.076 (4)	0.50
H35A	0.0679	0.3380	0.6891	0.114*	0.50
H35B	0.0562	0.2172	0.6730	0.114*	0.50
H35C	0.0933	0.2475	0.7318	0.114*	0.50
C36	0.0001 (3)	0.0690 (7)	0.7169 (4)	0.063 (3)	0.50
H36A	0.0370	0.0639	0.7090	0.075*	0.50
H36B	−0.0080	0.0032	0.7356	0.075*	0.50
C37	−0.0355 (8)	0.0791 (14)	0.6618 (8)	0.072 (5)	0.50
H37A	−0.0299	0.0175	0.6382	0.107*	0.50
H37B	−0.0271	0.1430	0.6416	0.107*	0.50
H37C	−0.0728	0.0814	0.6686	0.107*	0.50

### Atomic displacement parameters (Å^2^)

**Table:**

	*U*^11^	*U*^22^	*U*^33^	*U*^12^	*U*^13^	*U*^23^
Mn1	0.0194 (2)	0.0256 (3)	0.0178 (2)	0.00585 (18)	0.00654 (16)	0.0020 (2)
N4	0.0220 (11)	0.0259 (16)	0.0192 (13)	0.0031 (10)	0.0042 (9)	−0.0006 (11)
N5	0.0212 (10)	0.0234 (15)	0.0159 (12)	0.0017 (10)	0.0045 (9)	−0.0002 (11)
O1	0.0235 (9)	0.0327 (14)	0.0225 (11)	0.0096 (9)	0.0088 (8)	0.0039 (10)
O1W	0.0277 (10)	0.0267 (13)	0.0274 (12)	0.0059 (9)	0.0064 (9)	−0.0024 (10)
O2	0.0259 (9)	0.0271 (13)	0.0208 (11)	0.0100 (9)	0.0080 (8)	0.0017 (9)
O2W	0.0513 (13)	0.0386 (15)	0.0287 (13)	−0.0140 (11)	0.0126 (11)	−0.0040 (11)
O3	0.0313 (11)	0.0445 (16)	0.0281 (13)	0.0057 (10)	0.0161 (9)	0.0062 (11)
O4	0.0388 (11)	0.0376 (15)	0.0264 (12)	0.0155 (10)	0.0138 (10)	0.0135 (11)
C1	0.0197 (12)	0.0272 (19)	0.0248 (16)	0.0023 (12)	0.0047 (11)	−0.0058 (14)
C2	0.0246 (13)	0.035 (2)	0.0266 (17)	0.0023 (13)	0.0090 (12)	−0.0002 (15)
C3	0.0261 (15)	0.051 (2)	0.035 (2)	0.0054 (15)	0.0142 (14)	−0.0018 (18)
C4	0.0307 (16)	0.048 (2)	0.041 (2)	0.0207 (16)	0.0088 (14)	−0.0003 (18)
C5	0.0339 (16)	0.039 (2)	0.0325 (19)	0.0133 (15)	0.0064 (14)	0.0043 (16)
C6	0.0236 (13)	0.0289 (19)	0.0252 (17)	0.0069 (13)	0.0045 (12)	0.0012 (14)
C7	0.0258 (14)	0.0297 (19)	0.0229 (16)	0.0050 (13)	0.0029 (12)	0.0035 (14)
C8	0.0242 (13)	0.0249 (18)	0.0187 (15)	−0.0015 (12)	0.0073 (11)	−0.0006 (13)
C9	0.0287 (14)	0.035 (2)	0.0276 (18)	0.0065 (14)	0.0072 (13)	0.0059 (15)
C10	0.0444 (18)	0.036 (2)	0.0214 (17)	0.0023 (16)	0.0092 (14)	0.0069 (16)
C11	0.0357 (16)	0.031 (2)	0.0240 (17)	−0.0041 (14)	0.0141 (13)	0.0004 (15)
C12	0.0244 (13)	0.0280 (19)	0.0250 (17)	0.0000 (13)	0.0080 (12)	−0.0006 (14)
C13	0.0232 (13)	0.0204 (17)	0.0200 (15)	−0.0018 (12)	0.0049 (11)	−0.0003 (13)
C14	0.0219 (13)	0.0284 (19)	0.0207 (16)	0.0004 (12)	0.0086 (11)	−0.0032 (14)
C15	0.0231 (13)	0.0247 (18)	0.0229 (16)	0.0036 (12)	0.0035 (11)	−0.0002 (13)
C16	0.0281 (14)	0.041 (2)	0.0266 (17)	0.0130 (14)	0.0113 (13)	0.0017 (16)
C17	0.0327 (16)	0.039 (2)	0.041 (2)	0.0195 (15)	0.0107 (15)	0.0094 (18)
C18	0.0375 (16)	0.033 (2)	0.0331 (19)	0.0111 (15)	0.0084 (14)	0.0120 (16)
C19	0.0281 (14)	0.0280 (19)	0.0218 (16)	0.0046 (13)	0.0073 (12)	0.0026 (14)
C20	0.0201 (13)	0.0224 (18)	0.0203 (15)	0.0015 (12)	0.0038 (11)	−0.0012 (13)
C21	0.048 (2)	0.063 (3)	0.040 (2)	0.0018 (18)	0.0297 (17)	0.010 (2)
C22	0.062 (2)	0.051 (3)	0.038 (2)	0.020 (2)	0.0258 (18)	0.0244 (19)
Fe1	0.0182 (3)	0.0211 (4)	0.0228 (3)	0.000	0.0049 (2)	0.000
N1	0.0449 (16)	0.040 (2)	0.0385 (17)	−0.0147 (14)	0.0206 (14)	−0.0062 (14)
N2	0.0348 (15)	0.0362 (19)	0.055 (2)	0.0074 (13)	0.0125 (14)	−0.0030 (16)
N3	0.0594 (19)	0.048 (2)	0.0325 (18)	0.0101 (16)	−0.0060 (15)	−0.0024 (16)
C23	0.0257 (14)	0.031 (2)	0.0262 (17)	0.0000 (14)	0.0114 (12)	−0.0040 (14)
C24	0.0217 (13)	0.0251 (19)	0.0347 (18)	−0.0002 (14)	0.0057 (12)	0.0000 (15)
C25	0.0299 (15)	0.027 (2)	0.037 (2)	0.0068 (14)	0.0062 (14)	0.0025 (16)
O5	0.092 (2)	0.056 (2)	0.0462 (18)	−0.0256 (16)	0.0231 (15)	−0.0015 (15)
C26A	0.093 (9)	0.059 (5)	0.058 (4)	−0.022 (6)	0.034 (5)	−0.011 (3)
C27	0.130 (7)	0.105 (7)	0.079 (6)	0.012 (6)	0.066 (6)	−0.042 (5)
C26B	0.093 (9)	0.059 (5)	0.058 (4)	−0.022 (6)	0.034 (5)	−0.011 (3)
O6	0.0588 (15)	0.0536 (19)	0.0352 (15)	0.0273 (13)	0.0070 (13)	0.0013 (13)
C28A	0.042 (8)	0.074 (12)	0.080 (12)	0.023 (8)	−0.002 (7)	0.036 (8)
C29	0.105 (8)	0.145 (12)	0.052 (7)	−0.009 (8)	0.006 (6)	−0.035 (7)
C28B	0.041 (6)	0.038 (7)	0.031 (6)	−0.005 (5)	0.023 (5)	−0.020 (5)
N6	0.024 (4)	0.029 (3)	0.086 (15)	0.003 (4)	0.002 (9)	−0.004 (4)
C30	0.029 (4)	0.059 (7)	0.157 (11)	0.014 (4)	0.000 (5)	−0.003 (7)
C31	0.067 (7)	0.062 (9)	0.111 (12)	0.018 (6)	0.014 (9)	−0.021 (11)
C32	0.032 (4)	0.048 (7)	0.111 (10)	0.001 (4)	−0.007 (5)	−0.003 (6)
C33	0.070 (7)	0.068 (11)	0.118 (16)	0.002 (7)	0.010 (8)	0.057 (9)
C34	0.056 (8)	0.032 (5)	0.163 (16)	0.000 (4)	−0.018 (7)	0.015 (7)
C35	0.070 (7)	0.063 (9)	0.089 (11)	−0.031 (6)	−0.007 (8)	0.020 (9)
C36	0.051 (4)	0.028 (5)	0.107 (8)	−0.005 (4)	0.001 (5)	−0.003 (5)
C37	0.067 (9)	0.074 (16)	0.073 (11)	−0.019 (11)	0.006 (7)	−0.027 (10)

### Geometric parameters (Å, °)

**Table:**

Mn1—O2	1.880 (2)	Fe1—C23	1.953 (3)
Mn1—O1	1.884 (2)	Fe1—C25	1.958 (4)
Mn1—N5	1.996 (2)	Fe1—C25^i^	1.958 (4)
Mn1—N4	1.996 (3)	N1—C23	1.152 (4)
Mn1—O2W	2.210 (3)	N2—C24	1.145 (4)
Mn1—O1W	2.274 (2)	N3—C25	1.158 (4)
N4—C7	1.307 (4)	O5—C26A	1.386 (13)
N4—C8	1.434 (3)	O5—C26B	1.594 (7)
N5—C14	1.306 (4)	O5—H5B	0.8747
N5—C13	1.424 (4)	C26A—C27	1.679 (13)
O1—C1	1.326 (3)	C26A—H26A	0.9700
O1W—H1C	0.8500	C26A—H26B	0.9700
O1W—H1D	0.8500	C27—H27A	0.9600
O2—C20	1.328 (3)	C27—H27B	0.9600
O2W—H2C	0.8501	C27—H27C	0.9600
O2W—H2D	0.8499	C26B—H26C	0.9600
O3—C2	1.366 (4)	C26B—H26D	0.9600
O3—C21	1.444 (3)	C26B—H26E	0.9600
O4—C19	1.372 (3)	O6—C28A	1.33 (2)
O4—C22	1.434 (4)	O6—C28B	1.472 (16)
C1—C6	1.402 (4)	O6—H6B	0.8619
C1—C2	1.427 (4)	C28A—C29	1.40 (2)
C2—C3	1.382 (4)	C28A—H28A	0.9700
C3—C4	1.395 (5)	C28A—H28B	0.9700
C3—H3A	0.9300	C29—H29A	0.9600
C4—C5	1.370 (4)	C29—H29B	0.9600
C4—H4A	0.9300	C29—H29C	0.9600
C5—C6	1.425 (4)	C28B—H28C	0.9600
C5—H5A	0.9300	C28B—H28D	0.9600
C6—C7	1.437 (4)	C28B—H28E	0.9600
C7—H7A	0.9300	N6—N6^ii^	0.58 (3)
C8—C9	1.393 (4)	N6—C36^ii^	1.252 (11)
C8—C13	1.410 (4)	N6—C34^ii^	1.377 (11)
C9—C10	1.390 (4)	N6—C30	1.46 (2)
C9—H9A	0.9300	N6—C32	1.50 (3)
C10—C11	1.392 (4)	N6—C36	1.561 (15)
C10—H10A	0.9300	N6—C34	1.571 (12)
C11—C12	1.378 (4)	C30—C31	1.556 (18)
C11—H11A	0.9300	C30—H30A	0.9600
C12—C13	1.400 (4)	C30—H30B	0.9600
C12—H12A	0.9300	C31—H31A	0.9600
C14—C15	1.428 (4)	C31—H31B	0.9600
C14—H14A	0.9300	C31—H31C	0.9599
C15—C20	1.417 (4)	C32—C33	1.581 (14)
C15—C16	1.420 (4)	C32—H32A	0.9598
C16—C17	1.361 (4)	C32—H32B	0.9600
C16—H16A	0.9300	C33—H33A	0.9600
C17—C18	1.408 (4)	C33—H33B	0.9601
C17—H17A	0.9300	C33—H33C	0.9598
C18—C19	1.376 (4)	C34—C35	1.455 (16)
C18—H18A	0.9300	C34—H34A	0.9598
C19—C20	1.412 (4)	C34—H34B	0.9600
C21—H21A	0.9600	C35—H35A	0.9601
C21—H21B	0.9600	C35—H35B	0.9600
C21—H21C	0.9600	C35—H35C	0.9600
C22—H22A	0.9600	C36—C37	1.44 (2)
C22—H22B	0.9600	C36—H36A	0.9602
C22—H22C	0.9600	C36—H36B	0.9599
Fe1—C24^i^	1.945 (3)	C37—H37A	0.9601
Fe1—C24	1.945 (3)	C37—H37B	0.9600
Fe1—C23^i^	1.953 (3)	C37—H37C	0.9599
			
O2—Mn1—O1	91.62 (10)	C23^i^—Fe1—C23	90.40 (19)
O2—Mn1—N5	93.02 (10)	C24^i^—Fe1—C25	86.79 (14)
O1—Mn1—N5	175.24 (9)	C24—Fe1—C25	87.36 (13)
O2—Mn1—N4	175.15 (8)	C23^i^—Fe1—C25	89.52 (13)
O1—Mn1—N4	92.89 (10)	C23—Fe1—C25	96.47 (13)
N5—Mn1—N4	82.44 (10)	C24^i^—Fe1—C25^i^	87.36 (13)
O2—Mn1—O2W	91.82 (11)	C24—Fe1—C25^i^	86.79 (14)
O1—Mn1—O2W	92.74 (10)	C23^i^—Fe1—C25^i^	96.47 (13)
N5—Mn1—O2W	88.17 (10)	C23—Fe1—C25^i^	89.52 (13)
N4—Mn1—O2W	89.75 (11)	C25—Fe1—C25^i^	171.5 (2)
O2—Mn1—O1W	92.23 (10)	N1—C23—Fe1	176.0 (3)
O1—Mn1—O1W	94.28 (9)	N2—C24—Fe1	179.4 (3)
N5—Mn1—O1W	84.49 (9)	N3—C25—Fe1	173.8 (3)
N4—Mn1—O1W	85.66 (10)	C26A—O5—H5B	107.4
O2W—Mn1—O1W	171.79 (8)	C26B—O5—H5B	94.1
C7—N4—C8	122.1 (3)	O5—C26A—C27	100.3 (10)
C7—N4—Mn1	124.2 (2)	O5—C26A—H26A	111.7
C8—N4—Mn1	113.49 (18)	C27—C26A—H26A	111.7
C14—N5—C13	122.2 (2)	O5—C26A—H26B	111.7
C14—N5—Mn1	124.3 (2)	C27—C26A—H26B	111.7
C13—N5—Mn1	113.47 (17)	H26A—C26A—H26B	109.5
C1—O1—Mn1	128.92 (19)	C26A—C27—H27A	109.5
Mn1—O1W—H1C	104.2	C26A—C27—H27B	109.5
Mn1—O1W—H1D	108.6	H27A—C27—H27B	109.5
H1C—O1W—H1D	123.9	C26A—C27—H27C	109.5
C20—O2—Mn1	128.80 (18)	H27A—C27—H27C	109.5
Mn1—O2W—H2C	118.9	H27B—C27—H27C	109.5
Mn1—O2W—H2D	115.8	O5—C26B—H26C	109.5
H2C—O2W—H2D	122.5	O5—C26B—H26D	109.5
C2—O3—C21	117.9 (2)	H26C—C26B—H26D	109.5
C19—O4—C22	116.9 (2)	O5—C26B—H26E	109.5
O1—C1—C6	124.4 (2)	H26C—C26B—H26E	109.5
O1—C1—C2	116.9 (3)	H26D—C26B—H26E	109.5
C6—C1—C2	118.7 (3)	C28A—O6—H6B	114.3
O3—C2—C3	125.5 (3)	C28B—O6—H6B	115.0
O3—C2—C1	114.2 (3)	O6—C28A—C29	123.8 (17)
C3—C2—C1	120.3 (3)	O6—C28A—H28A	107.4
C2—C3—C4	120.5 (3)	C29—C28A—H28A	107.1
C2—C3—H3A	119.8	O6—C28A—H28B	104.2
C4—C3—H3A	119.8	C29—C28A—H28B	105.6
C5—C4—C3	120.6 (3)	H28A—C28A—H28B	107.9
C5—C4—H4A	119.7	C28A—C29—H29A	109.5
C3—C4—H4A	119.7	C28A—C29—H29B	109.5
C4—C5—C6	120.1 (3)	H29A—C29—H29B	109.5
C4—C5—H5A	119.9	C28A—C29—H29C	109.5
C6—C5—H5A	119.9	H29A—C29—H29C	109.5
C1—C6—C5	119.8 (3)	H29B—C29—H29C	109.5
C1—C6—C7	123.2 (3)	O6—C28B—H28C	109.5
C5—C6—C7	117.0 (3)	O6—C28B—H28D	109.5
N4—C7—C6	126.2 (3)	H28C—C28B—H28D	109.5
N4—C7—H7A	116.9	O6—C28B—H28E	109.5
C6—C7—H7A	116.9	H28C—C28B—H28E	109.5
C9—C8—C13	119.7 (2)	H28D—C28B—H28E	109.5
C9—C8—N4	125.4 (3)	C30—N6—C32	114.0 (11)
C13—C8—N4	114.9 (2)	C30—N6—C36	109.9 (16)
C10—C9—C8	119.8 (3)	C32—N6—C36	107.9 (12)
C10—C9—H9A	120.1	C30—N6—C34	110.3 (12)
C8—C9—H9A	120.1	C32—N6—C34	106.2 (15)
C9—C10—C11	120.5 (3)	C36—N6—C34	108.4 (10)
C9—C10—H10A	119.8	N6—C30—C31	115.5 (11)
C11—C10—H10A	119.8	N6—C30—H30A	108.5
C12—C11—C10	120.2 (3)	C31—C30—H30A	107.3
C12—C11—H11A	119.9	N6—C30—H30B	108.1
C10—C11—H11A	119.9	C31—C30—H30B	109.6
C11—C12—C13	120.2 (3)	H30A—C30—H30B	107.5
C11—C12—H12A	119.9	C30—C31—H31A	109.6
C13—C12—H12A	119.9	C30—C31—H31B	109.3
C12—C13—C8	119.6 (3)	H31A—C31—H31B	109.5
C12—C13—N5	124.8 (3)	C30—C31—H31C	109.6
C8—C13—N5	115.6 (2)	H31A—C31—H31C	109.5
N5—C14—C15	126.1 (2)	H31B—C31—H31C	109.5
N5—C14—H14A	117.0	N6—C32—C33	115.0 (14)
C15—C14—H14A	117.0	N6—C32—H32A	109.9
C20—C15—C16	119.5 (3)	C33—C32—H32A	108.8
C20—C15—C14	123.6 (3)	N6—C32—H32B	108.0
C16—C15—C14	116.9 (3)	C33—C32—H32B	107.5
C17—C16—C15	120.5 (3)	H32A—C32—H32B	107.3
C17—C16—H16A	119.8	C32—C33—H33A	109.4
C15—C16—H16A	119.8	C32—C33—H33B	108.8
C16—C17—C18	120.5 (3)	H33A—C33—H33B	109.5
C16—C17—H17A	119.8	C32—C33—H33C	110.2
C18—C17—H17A	119.8	H33A—C33—H33C	109.5
C19—C18—C17	120.0 (3)	H33B—C33—H33C	109.5
C19—C18—H18A	120.0	C35—C34—N6	116.3 (11)
C17—C18—H18A	120.0	C35—C34—H34A	110.6
O4—C19—C18	124.8 (3)	N6—C34—H34A	108.8
O4—C19—C20	114.1 (2)	C35—C34—H34B	106.2
C18—C19—C20	121.1 (3)	N6—C34—H34B	107.1
O2—C20—C19	118.0 (2)	H34A—C34—H34B	107.5
O2—C20—C15	123.6 (3)	C34—C35—H35A	109.5
C19—C20—C15	118.3 (3)	C34—C35—H35B	107.2
O3—C21—H21A	109.5	H35A—C35—H35B	109.5
O3—C21—H21B	109.5	C34—C35—H35C	111.7
H21A—C21—H21B	109.5	H35A—C35—H35C	109.5
O3—C21—H21C	109.5	H35B—C35—H35C	109.5
H21A—C21—H21C	109.5	C37—C36—N6	116.3 (13)
H21B—C21—H21C	109.5	C37—C36—H36A	108.9
O4—C22—H22A	109.5	N6—C36—H36A	107.4
O4—C22—H22B	109.5	C37—C36—H36B	108.9
H22A—C22—H22B	109.5	N6—C36—H36B	107.9
O4—C22—H22C	109.5	H36A—C36—H36B	107.2
H22A—C22—H22C	109.5	C36—C37—H37A	107.7
H22B—C22—H22C	109.5	C36—C37—H37B	110.3
C24^i^—Fe1—C24	92.78 (19)	H37A—C37—H37B	109.5
C24^i^—Fe1—C23^i^	88.50 (14)	C36—C37—H37C	110.4
C24—Fe1—C23^i^	176.55 (13)	H37A—C37—H37C	109.5
C24^i^—Fe1—C23	176.55 (13)	H37B—C37—H37C	109.5
C24—Fe1—C23	88.50 (14)		

Symmetry codes: (i) −*x*+1, *y*, −*z*+3/2; (ii) −*x*, *y*, −*z*+3/2.

### Hydrogen-bond geometry (Å, °)

**Table:**

*D*—H···*A*	*D*—H	H···*A*	*D*···*A*	*D*—H···*A*
O1W—H1C···O3^iii^	0.85	2.14	2.959 (4)	162
O1W—H1C···O1^iii^	0.85	2.37	2.948 (3)	125
O1W—H1D···O4^iii^	0.85	2.14	2.929 (3)	153
O1W—H1D···O2^iii^	0.85	2.25	2.901 (3)	134
O2W—H2C···O6^iv^	0.85	1.88	2.698 (4)	160
O2W—H2D···O5^v^	0.85	1.91	2.751 (4)	168
O5—H5B···N3	0.87	2.08	2.934 (5)	166
O6—H6B···N2	0.86	1.88	2.739 (4)	173

Symmetry codes: (iii) −*x*+1/2, −*y*+1/2, −*z*+2; (iv) −*x*+1/2, *y*−1/2, −*z*+3/2; (v) *x*−1/2, *y*−1/2, *z*.
